# Indents

**Published:** 1917-12

**Authors:** 


### INDENTS

**☞ Brief Articles of Technical or Scientific Value will receive notice and comment. Contributions invited.**

*His Intention*

"Rastus," inquired the colonel, "aren't you ready to die for your country?"

"No, sah, Ah ain't studyin' to die foh mah country. Ah's studyin' to make some German die foh his country."

*Hospital Dental Service*

Great credit is due the medical profession for the development and wonderfully efficient organization of the modern hospital. It is but a comparatively short time since patients sought hospital accommodation only because they could not provide proper home treatment, and frequently looked upon such an event as a most unfortunate circumstance and tantamount to surrendering their last chance of life. How different today? Even the more wealthy citizen of the community voluntarily chooses hospital service, rather than home treatment, because of the possibility of a more efficient management of their case and the strong probability of an earlier convalescence. In other words, an institution, originally conducted for the benefit of the poor, has proved so advantageous in the treatment of disease, that the well-to-do-members of society have, in case of sickness, come to take full advantage of its many facilities. A spirit of carping criticism and public prejudice, against the hospital, has given place to whole-hearted co-operation, general commendation and generous public support.

Advantage has accrued to the community through the enlarged opportunity for clinical observation which hospitals afford, and the means whereby both medical students and nurses receive a more thorough and practical training.

This latter feature of hospital work has encouraged the establishment of research and experimental laboratories and the organization of pathological department which are of inestimable value to the physician. Adequate X-ray, hydro and electro therapy apparatus are also made readily available The hospital, as an institution, certainly stands today as the most perfect modern example of the application of the principles of "the healing art"

How can we conserve the best interests of the patient suffering from arthritis or other condition, caused by foci of infection lodged about the roots of teeth, unless there be the closest possible cooperation between physician and dentist? And how can we have that cooperation without a dental service, organized along similar lines to the other services or departments of the hospital? The writer has frequently heard of hospitals where dentists were invited to organize a dental service, but where the invitation was neglected or ignored. There are, surely, dentists in every community who stand ready to cooperate with the hospital authorities and who will gladly give their time gratuitously to the operation of such a service. We are sometimes apt to complain that physicians know too little about dentistry, but after all, have dentists a relative knowledge of medicine? The time is past for such comparisons. *In the interests of the public, cooperation is the immediate necessity.* Medical and dental science would be appreciably advanced thereby, and much of the suffering of humanity greatly alleviated or entirely prevented.

This is surely the "next forward step" and the members of the dental profession, in every community, should see, that in so far as they are able, the highest possible standard of dental efficiency is maintained in their local hospital and that this service is rendered in such a generous and scientific manner, that credit will redound to the dental profession. We can conceive of no greater stimulus for dentistry than this, and believe there would be an immediate response upon the part of the profession and an earnest striving to make the dentistry of tomorrow, more worthy of the place it now occupies in the field of public health.—Editorial in *Oral Health*.

*Aluminum Clasps* Teeth embraced by gold clasps undoubtedly, sooner or later, show signs of decay. After many experiments we have resolved to always use aluminum clasps in the future. On examination of teeth upon which aluminum clasps have been worn for considerable time, we have quite failed to find the slightest sign of decay or erosion.—*Oral Health,* for October, 1917.

*Plaster Models* Many good models are spoiled by placing the wet bite back on the model. Dry the wax with a doily or compressed air, each time you take it from the mouth wet with saliva, and you will not spoil the surface of your plaster models, and your plates will fit better.—Homer Almon, *Dental Review.*

*A Dangerous Injury* Commercial Traveler: That man over there says the Government owes him $6,000 back pension. How does he make it out?

Grocer: Hanged if I know! As near as I can figure it out, he had his retreat cut off at Gettysburg.—*Puck.*

*Why Teeth Decay* Clean teeth seldom decay. If the first set of teeth be properly cared for the roots will be absorbed normally, allowing each permanent tooth to take its rightful place in the mouth. If these in turn are kept clean by mechanical means, very few cavities develop, and the only way for them to give out is by the actual wearing away of the grinding surfaces. Not many people live long enough to wear out their teeth.

Our children are fed on soft, starchy foods, which require little chewing, and hence no mechanical cleansing. The gums become soft and flabby. As the teeth come through, particles of this easily decomposed food collects around and upon them, and soon become masses of acid-forming bacteria.

If these were removed at once they would do no great harm, but being allowed to remain, the acid attacks the enamel of the tooth, partially dissolving it, and a cavity is formed.

Once formed, this cavity collects food particles and more bacteria, producing more acid, which dissolves more of the enamel; this makes a larger cavity, which collects more food, bacteria, etc., until the pulp or nerve of the tooth is reached, and the child is brought with the toothache to the dentist.

One tooth after another becomes affected; some are extracted; others are so badly decayed that when the permanent teeth begin to form, they are deflected from their proper places. This causes crooked teeth, and the services of a specialist are necessary to correct malformations of the jaws and nasal cavity. The bacteria from the baby teeth are soon transmitted to the second teeth, and very often these are badly decayed before they are quite through the gums.

Parents who are not satisfied with the growth and development of their children, bodily, mentally, or both, should examine carefully the condition of the mouth and teeth. If the teeth are found decayed, out of line, or in any other than perfect condition, the chances are ninety times in every hundred, that the cause of the unsatisfactory condition of the child lies right there, and should be remedied at once. Taken in time, the remedy is easy and sure; and no parent has the right to condemn a child to go through life handicapped by conditions that are so easily corrected. —*Oral Health*, for November.

*For Spongy Gums and Inflamed Membrane*

℞ Potass. chloratis, ʒ i j; sodium diboratis, ʒ j; potass. nitratis, ʒ s s; tinct. arnica, ʒ i j; aq. cinamomi—q. s.-ad., ℥ v i i j M. Sig.—Mouth wash as directed T. I. D. If used after each meal, full strength, this will work out beautifully.—J. C. Schmitt, *Dental Summary.*

*He Was Presumably Going South*

While putting out street fires one Home Defense Leaguer was killed, another was knocked senseless with his own night stick and a third was painfully cut on the west side last night.—New York *Mail.*

*Brief Outline of Technic for Root Amputation*

Not only instruments, but the operator's bracket handpiece should be thoroughly sterilized before operating. The oral mucosa which is always infected, must be thoroughly sterilized. Iodin occupies the predominate place among medicinal agents. Benzine is also used, and a combination of iodin and aconite. These reagents are indispensable and are destructive to superficial bacteria. They also produce dryness of mucosa, tanning of the skin, fixing the bacteria for some time in such a way that they are quite dormant, if not destroyed. The iodin besides these properties, retards bacterial growth, by clinging for a long time to the tissue to which it has been applied, as low as 1 to 8,000 has been known to retard the bacterial development. Not only should the iodin be applied, but it should be repeatedly rubbed back and forth, mechanically cleansing the surface.

As the injection generally precedes surgical intervention it plays a part in the subsequent healing of the wound. For this reason even in a very minute operation, full attention should be given to asepsis as untoward sequelæ may arise from neglect of one of the factors involved. Many cases of edema following injection are attributed to insufficient asepsis, such as: neglect in sterilization of the hands, instruments, and other things really brought in contact with field of operation.

Radiography is indispensable in root amputation, not only as a guide, but it establishes the extent of the osteomyelitis.

Local anesthetics most often used should be boiled, making the solution sterile.

In making the operation a one-half to three-quarters inch transverse incision is made approximately two millimeters from the apex, or toward crown of the tooth, or better, incision should be made in the middle of field of operation. At times it is necessary to ligate small vessels to stop hemorrhage. In the majority of cases application of sterile swabs will accomplish the same results. The periosteum should now be gently pushed out both ways from incision, and retractors used; now the eminence over the root of tooth can be plainly seen, the radiograph having established the extent of the area involved and the position most suitable for entrance to area.

The external plate and spongy process over the roots of the eight anterior teeth vary from one-half millimeter to two millimeters. Thoroughly expose the root with small chisels (mastoid chisels). With a fissure bur, cut across the root at incisal end of area involved; remove the part of the root severed, then thoroughly curette diseased bone; with small files round up the end of tooth, then a hot instrument should be used to sear the guttapercha root filling. In the majority of cases the two flaps of wound will approximate when the retractors are released. But where there is extensive osteomyelitis both ends should be stitched and an iodoform gauze stringer left in such a manner that it can be partly removed each day for about three days.—R. C. Lane, in *The Dental Items of Interest.*

*Bite Testing* Have the compound on the occlusal surface very soft, insert the impression to place and instruct the patient to "close the lips and swallow." Do not in any way suggest biting because if the patient brings the jaws together without thinking of biting, the condyles will remain in their normal position in the fossæ and the normal relation of the opposing arches will be accurately recorded.

Now trim off all the compound on the occlusal where the teeth have imbedded themselves into the soft compound, leaving only the very tips of the cusps showing. Place the impression again to place and have the patient snap the lower jaw up against the upper compound several times in quick succession. If the bite is correct the patient will be able to strike the lower teeth in their corresponding depressions each time. If, however, the bite is not correct, the teeth will not fit their corresponding depressions and it will be necessary for accuracy to take the bite again by warming up the occlusal surface and again having the patient close the lips and swallow.—Bradley F. Lockwood, in *American Dentist.*

*Oral Hygiene Regieme* Some years ago it occurred to me that the operator who was called upon to put a mouth in a hygienic condition should have a systematic set of rules of procedure, and the understanding that the fundamental law of mouth hygiene is the same as "remove the cause, is the first law of surgery."

First: To put a mouth in a hygienic condition consists of the removal of all deposits by surgical means, the removal of all stains by polishing with engine brushes, porte polishers and tape. Scaling and polishing the teeth should be the initial operation. Many think mouth hygiene consists only of this operation, and when it is completed often in a superficial and an indifferent way, many times left until all other work in the mouth is finished or turned over to an assistant, that their duty here ends.

Second: The removal of all septic irritants, such as overhanging fillings, ill-fitting crowns and treatment and cure of diseased mucous tissue.

Third: The extraction of roots, incurable abscessing teeth and in the majority of cases of the third molars, especially in crowded arches, for impacted third molars in many cases cause criminal development and tendencies, moral degeneracy and nervous irritation as do adenoids and mouth breathing. Anti-tobacconists made much of the death of Gen. U. S. Grant being caused by cancer from the use of tobacco; however, the initial cause was a jagged third molar root that caused the cancer that ended his career.

Fourth: The ligation of loosened teeth under treatment, with 28-gauge wire. The removal of carious tooth tissue, medication and filling of cavities temporarily with guttapercha, and later the proper restoration of the same, remembering the observing operator always anticipates caries.

Fifth: The restoration of missing teeth with partial dentures, crowns or bridges.

Sixth: The thorough instruction of the patient at the chair in proper home prophylaxis, in the use of brush, dentifrices, tongue-scraper, tape and gum massage and the insistance on stated periodic visits for examinations and treatments.—B. L. Thorpe, in *Dental Summary*.

*Use of Suprarenin*

Regarding the use of suprarenin as an addition to a local anesthetic solution, we should be extremely guarded relative to the quantity of this highly reactive compound. Ordinarily, we add one drop of the suprarenin solution (1-1000) to one cubic centimeter of the anesthetic solution. But as we increase the quantity of the solution to be employed for a single injection, we must proportionally reduce the amount of suprarenin. Usually we employ it according to the following graded schedule: Suprarenin, one drop added to 1 c.c. anesthetic solution; two drops added to 3 c.c. of solution; three drops added to 5 c.c. of solution; four drops added to 8 c.c. of solution; five drops added to 10 c.c. of solution.

If we employ the suprarenin in the above proportions we avoid to a large extent the toxic effects which are prone to occur if we use it in too large quantities.—Herman Prinz, in *Dental Cosmos.*

*Sulpho-Carbolate of Zinc* The best remedy the speaker has yet discovered to aid in conjunction with gum massage is the formula of Dr. Will H. Whitslar known as "zinc sulpho-carbolate:" ℞ Zinc sulpho-carbolate, 60 grains; alcohol, 1 ounce; distilled water, 2 ounces; oil wintergreen, 8 drops. This for curative action and hardening the gums is excellent.—B. L. Thorpe, in *Dental Summary.*

*Taking Impressions With Composition* When the compo is moulded properly on the tray, immerse the external aspect of the tray for a few minutes in cold water (this prevents the heated metal causing discomfort and sometimes pain to the lips), dry and dust surface of compo with fine quality French chalk and take your impression. This method gives very fine definition, the compo does not stick to the teeth, and the general result is a source of pleasure to the dentist and his patient.

*Concerning Cancer* Cancer is not a "blood disease," but always starts as a local affair; hence it can always be cured by removal if discovered and treated early enough.

Cancer, in the beginning, may cause no pain or other symptoms of ill-health.

Cancer is probably not hereditary.

Cancer is not contagious.

No up-to-date doctor will treat a condition that might mean cancer without thorough examination.

The cancer patient must learn to seek treatment as promptly as a patient with appendicitis.

Precancerous conditions can be detected in the mouth eight years prior to the operative stage. If suspected, decayed tooth structure and debris removed from outside of teeth should be submitted to the bacteriologist at once for examination.

*To Increase Efficiency of Local Anesthetic Solutions* The addition of adrenalin chlorid (20 drops of the 1-100 Sol. to the ounce of local anesthetic solution) does three things: 1. Renders the operative field comparatively bloodless. 2. Greatly intensifies and prolongs the anesthetic effect. 3. Renders the solution much less toxic by retarding its absorption into the systemic circulation.—P. G. Puterbaugh, in *Dental Review.*

*Doctors of Dental Surgery* I would like to ask a question. Do we deserve to be called doctors? ''Of course,'' I hear the chorus answer, ''Are we not a branch of the medical profession? Do we not ease pain and relieve suffering?'' I know all of that, but really is there so very much more virtue in plugging a tooth in the human anatomy than a leaky gas pipe? The olfactory nerve is just as important as the trigeminal and I think it can be said without any fear of contradiction that the cleaning of a sewer is as necessary to health as the cleaning of the mouth, yet the gas fitter or plumber is not called doctor. ''Sure,'' some will interpose, ''those are trades, but we are a learned profession.'' I admit we are a learned profession; so are the engineers, the teachers, the chemists, yet the people do not generally address them as doctors.

I may be wrong, but I think the reason for the special honor conferred by society upon the physician is not for what he knows or for the particular services he renders, but rather for what he is. Others know as much as the physician and more. The cobbler, the baker and the candlestick maker are as useful and necessary in the social state as the doctor, but in no other body of men, in no other calling, does the individual give so much of himself and give so freely and liberally. Poor people may have to go without food or clothing or dentistry, but not without medical attention. No one gives as much time and labor for nothing as the doctor—no matter how busy he may be or how much demand there is for his services, a brother practitioner or his family is always treated gratis. The medical man has nothing to sell to his fellows. He does not give them courses at fabulous fees and his ethical standard is above that of the average calling and he more than lives up to it. Compared with him we are mere tradesmen, our standard is not far above that of the honest grocer and therefore in my opinion the title ''doctor'' is wholly undeserved. I know some dentists will get very indignant, or even sore, over this statement, because the truth always hurts. However, I am quite willing to be shown. I am open to conviction and will gladly apologize to you—one and all—if proven wrong. Only, please remember, if you are after my hide, be sure that you are armed with facts, otherwise you will not get it. Generalities do not count and incidents do not prove a truth.—Editorial in *American Dentist.*

*Extracted Teeth to Be Replaced Immediately After Extraction*

The psychological moment to induce the patient to have a bridge replacement is at the time a tooth is extracted. They are either condemning their own negligence or the carelessness of some dentist whom they regard as responsible for their loss. Furthermore, the loss is more keenly felt than it will probably ever be again. If they are told by the operator (as they usually are) to wait from six weeks to six months for resorption to take place before having the substitute inserted, they may decide that they do not need it very much anyway, nor can we particularly blame them.

*I believe that the dentist's advice to wait for resorption is responsible for a great majority of the cases which we see every day where a tooth has been out for several months or years, and with the resultant wreckage of the occlusion on one side,* if not the whole upper and lower arches. On the contrary, the patient should be informed that the bridge can and should be immediately inserted, in fact, as in a case of pyorrhea, can be actually made previous to the extraction of the offending tooth and inserted at the same sitting. *By immediate insertion of a bridge the liability of any untoward occlusal damage in the future is averted,* to say nothing of avoiding pathological complications of the gingiva of the malpósed teeth. The immediate insertion of bridges can be accomplished with unvarying success by the employment of the *detachable saddle.* When this procedure is employed, it is necessary to readjust the saddle two or three times within the first six months or a year. If the method suggested by Dr. Carl D. Lucas, of Indianapolis, is followed at the time of extracting the tooth, I believe that one or two readjustments of saddle may be eliminated.—A. L. Van Arsdall, D.D.S., in *The Western Dental Journal.*

*The Effects of Organic Acids upon the Teeth* This note is barely more than a reiteration of Pickerill's well-known views on this topic. The main action of organic acids in the right strength and quantity, in the oral cavity is as salivary stimulants. To be accurate, it is not the effect of organic acids on the teeth which must be considered, but the effect of organic acids plus stimulated saliva on the teeth.

An appended table, summarizing a few experiments, shows a marked increase in the alkalinity and amylolytic power per minute for the saliva following the ingestion of fruits—banana, orange, apple—over the alkalinity and amylolytic power of "resting" saliva and saliva poured out after the eating of bread and butter or cake.

A point which finds repeated insistence is that the question of dosage is of extreme importance.

The resistance of the teeth varies enormously. Of this Pickerill is convinced, despite the usual deductions from the work of Tomes and Black. It is the *physical* and not the chemical consistence which is the essential thing in determining the resistance of teeth to acids. Instruments of precision indicate that teeth vary in hardness and in density. Weak acids indicate that teeth vary in solubility, and silver nitrate indicates that teeth vary in permeability. These differences are partly developmental and partly acquired.

These are points which find expression in Pickerill's early work, and are re-emphasized on this occasion to correct certain misconceptions which have arisen among his critics.—*Dental Cosmos.*

*Impression Help* When making a maxillary impression of an edentulous case with modeling compound, it seems as though we were going to be defeated in getting it to stick. After all the details have been taken care of, very carefully heat the rim and posterior border slightly, introduce into the mouth with care and carry it to place. Now instruct the patient to suck as it were on a straw immersed in syrup. This muscular and tongue effort on the part of the patient will turn the trick as nothing else can. The results are most satisfying.—Dr. Frahm, in *Pacific Dental Gazette.*

*Be Thorough* Many times a dentist will glance into a mouth, and in a few minutes lay out a plan for its complete repair. This is wrong, because in dentistry a hastily made plan often proves erroneous and costly for patient and dentist. The proper way is to make a careful and thorough examination, study models and X-ray pictures, and then through careful deliberation, reach a conclusion. In dentistry, at present, there is too much execution and not enough thought. Perhaps if we used more thought we would have less executing to do.—O. de F. Davis, *Dental Review.*

*An Old Anesthesia* A British gunner, who had successfully passed a blacksmith's course, was home on a furlough, wearing the hammer and pincers on his arm, when he was accosted by a civilian, who asked what the decoration was for.

"Oh," replied Tommy, "I'm an army dentist!"

"I see," said the civilian. "Of course, the pincers are for extracting teeth. But what is the idea of the hammer?"

"Well, you see, it's like this. Some of the chaps are a bit nervous, so we use the hammer to chloroform them," was the reply.

*Contributory Causes of Cancer of the Throat* Among the most constant conditions present in cases of cancer of the throat is sepsis of the throat from dental caries, pyorrhea alveolaris, or septic gingivitis. Normally the oral fluids are alkaline in reaction, but in the presence of these septic conditions they become acid. The increased irritating effects of such acid fluids on the mucosa of the throat is considerable, and may well prove a contributory factor to cancer of the throat.—Dr. W. S. Low, *Cosmos.*

*Salesmanship in Dentistry* Study your patient. Study the mouth conditions thoroughly, make a plan of how it can best be restored. Then don't try to sell the plan or the restoration of the mouth or, in fact, anything at all. You have nothing for sale, but get the patient's absolute confidence in your ability to take care of the case competently. Or in other words, have them, or get them in a frame of mind so they will utter words, as for example, "Doctor, I am in your hands, take care of my mouth as you would have your own taken care of." This, in a nutshell, is "Salesmanship in Dentistry."—W. O. Fellman, *Dental Review*.

